# Assessment of Adolescents in Child-to-Parent Violence: Invariance, Prevalence, and Reasons

**DOI:** 10.3390/children11070845

**Published:** 2024-07-12

**Authors:** Luis Burgos-Benavides, M. Carmen Cano-Lozano, Andrés Ramírez, Samuel P. León, Venus Medina-Maldonado, Francisco Javier Rodríguez-Díaz

**Affiliations:** 1 Department of Psychology, Universidad de Oviedo, 33003 Oviedo, Spain; burgosluis@uniovi.es (L.B.-B.); aramirezc1@ups.edu.ec (A.R.); gallego@uniovi.es (F.J.R.-D.); 2 Department of Psychology, Universidad de Jaen, 23071 Jaen, Spain; mccano@ujaen.es; 3 Department of Clinical Psychology, Universidad Politécnica Salesiana, Cuenca 010107, Ecuador; 4 Department of Pedagogy, Faculty of Humanities and Education Sciences, University of Jaen, 23071 Jaen, Spain; sparra@ujaen.es; 5 Centro de Investigación para la Salud de América Latina (CISeAL), Nursing Faculty, Pontificia Universidad Católica del Ecuador, Nayón 170530, Ecuador

**Keywords:** child-to-parent violence, questionnaire, adolescents, invariance measure, prevalence

## Abstract

Child-to-parent violence is a form of family violence where the children are the aggressors. Objective: This study first aimed to analyze the psychometric validity of the Child-to-Parent Violence Questionnaire (CPV-Q) among Ecuadorian adolescents. Second, the measurement invariance across the children’s sex was examined. Finally, the prevalence of child-to-parent violence (CPV) was also determined. Methods: A total of 2084 adolescents aged 12 to 18 years participated in this study. The participants were residents of two provinces in southern Ecuador. A higher percentage of participants were women. They lived mostly with their father and mother. The married marital status of the parents was the most frequently reported. Most of the fathers and mothers of the participants completed primary education. The sampling design was probabilistic, with proportional allocation by age and quotas according to the number of inhabitants per province. The sample size was determined by using a finite population formula. All the participants were randomly selected. The CPV-Q was used to assess child-to-parent violence. The items were adapted linguistically and tailored to the Ecuadorian context. Results: The questionnaire demonstrated excellent model fit and adequate reliability. Full measurement invariance was held for all scales except for the reasons subscale pertaining to fathers. Statistically significant differences were observed with respect to the sex of the aggressor, with control/domain behaviors toward both parents and psychological violence toward the mother being more frequent among daughters than sons. Similarly, reactive reasons were more frequent among daughters than sons. Conclusion: This study provides significant psychometric evidence on the CPV-Q in Ecuadorian adolescents along with data on the prevalence of violence against parents.

## 1. Introduction

Over the past decade, child-to-parent violence (CPV), a complex issue in which children assume the role of perpetrators, has been explored with particular interest. Despite being the least studied type of violence in comparison to other types of family violence [1], in recent years, this social problem has experienced an exponential increase in international research [2]

CPV is violence in which children perpetrate acts of physical, psychological, or financial abuse to control and domain their parents [3,4]. This excludes occasional violence and violence that occurs in states of diminished consciousness, drug withdrawal syndromes, delusional states, or parricides without a documented history of aggression [5].

Despite a significant increase in CPV research, its prevalence remains unclear [6], with highly variable figures depending on study type. These limitations in estimating prevalence are common to other forms of family violence [7,8]. Generally, the prevalence of psychological violence ranges from 28.8% to 94.7%, physical violence from 2.5% to 25%, financial violence from 60%, and control/domain behaviors from 70% [9,10].

The importance of studying these rates lies in providing epidemiological evidence and in knowing aspects for the prediction of violent behavior, the influence of parental attributions, or other risk factors for the violent behavior of children. Therefore, the assessment of CPV is a key aspect that requires reliable and valid measures [11,12].

Ibabe [12] identified 11 instruments with evidence of psychometric validity for assessing CPV, three of which have been utilized in Latin American samples. The commonly used measures in Mexico include the Child-to-Parent Aggression Questionnaire [13,14,15,16,17] and Conflict Tactics Scale [18,19]. In Chile, researchers have employed the Child-to-Parent Violence Questionnaire, adolescent and youth version [20,21], and the Child-to-Parent Aggression Questionnaire [22,23].

Previous studies have examined the psychometric properties of the Child-to-Parent Violence Questionnaire (CPV-Q) in adolescent samples (File S1). Contreras [24] used a Spanish sample and found adequate construct validity and internal consistency for four factors (psychological, physical, financial, and control/domain violence). Similarly, Jiménez-García [20], utilizing a Chilean sample, corroborated the four-factor structure with satisfactory fit indices and factor loadings for both the parental scales. Although these findings demonstrate the utility of CPV-Q, additional evidence is warranted across diverse cultural contexts. Recent studies have investigated psychometric instruments and models to explain the risk factors associated with CPV [25].

The present study provides evidence regarding the applicability of the CPV-Q to Ecuadorian adolescents. Given the scarcity of research on CPV in Ecuador, this study had three primary objectives: The first objective was to adapt and analyze the evidence for the psychometric validity of the Child-to-Parent Violence Questionnaire (CPV-Q) in an Ecuadorian sample. The second objective was to demonstrate the measurement invariance of CPV according to the child’s sex. The third goal was to examine the prevalence of different types of CPV (psychological, physical, financial, and control/domain) perpetrated toward mothers and fathers.

The following hypotheses were formulated based on the extant scholarly literature:

**Hypothesis** **1:** *We expected to confirm the factor structure of the CPV-Q and demonstrate optimal reliability, along with adequate discriminant and convergent validity. Specifically, we hypothesized that the instrument would identify factors of psychological, physical, financial, and control/domain violence. Despite being the least examined factor, the control/domain encapsulates coercive, manipulative behaviors to damage and exert control over parents. Multiple theoretical frameworks underscore the value of assessing this dimension when evaluating CPV. The literature situates CPV-Q among the top three measures available for evaluating this phenomenon [12].*

**Hypothesis** **2:** *We expected the CPV-Q to exhibit invariance as a function of the child’s sex. While some studies have reported sex differences in CPV manifestations, many have not explicitly tested the measurement invariance of the psychometric instruments used. Establishing invariance would provide further evidence that observed sex differences represent true distinctions in latent traits [26]. Assessing invariance is imperative for determining whether the CPV-Q measures the same constructs similarly for both sons and daughters rather than reflecting differential item functioning.*

**Hypothesis** **3:** *We expected no differences in CPV-Q scores according to the sex of the children for any of the CPV types. We did not expect to find differences in scores for instrumental reasons, but we expected to find that daughters obtain higher scores than sons for reactive reasons [20].*

## 2. Materials and Methods

### 2.1. Participants

The sample comprised 2084 participants: 56.4% girls (*n =* 1176) and 43.60% boys (*n =* 908) between the ages of 12 and 18 years (*M* = 14.38; *SD* = 1.62). The majority were Ecuadorians (98.2%, *n =* 2047). The participants resided in the Ecuadorian provinces of Azuay (62.5%, *n =* 1303) and Cañar (37.5%, *n =* 781), located in the south of the country. Regarding living arrangements, 52.6% (*n =* 1097) of the participants resided with their mothers and fathers, while 31% (*n =* 646) lived with their mothers. Additionally, 5.5% (*n =* 114) lived with their mothers and new partners, 7.6% (*n =* 158) lived with grandparents or other relatives, 2.1% (*n =* 44) lived with only their fathers, and 1.2% (*n =* 25) lived with their fathers and new partners.

Regarding parental marital status, 52.7% (*n =* 1099) were married, 22.5% (*n =* 469) were divorced or separated, 10.5% (*n =* 219) had never lived together, 9.9% (*n =* 207) were unmarried but cohabited, and 4.3% (*n =* 90) were widowed. Finally, 40.6% (*n =* 847) of fathers and 49.2% (*n =* 1025) of mothers had an educational level up to primary school, 20.8% (*n =* 434) of fathers and 24.6% (*n =* 512) of mothers had an education level up to secondary school, 8.1% (*n =* 161) of fathers and 9% (*n =* 188) of mothers had education at the university level, 6.2% fathers (*n =* 130) and 8.8% (*n =* 184) of mothers did not have any level of education, and 4.6% (*n =* 96) of fathers and 5.3% (*n =* 111) of mothers had completed postgraduate degrees.

### 2.2. Measures

#### 2.2.1. Ad Hoc Sociodemographic

An ad hoc form was administered to collect sociodemographic data, including age, sex, parental marital status, and parental education level.

#### 2.2.2. Child-to-Parent Violence Questionnaire (CPV-Q), Adolescent Version

The CPV-Q was developed by Contreras [24]. It assesses CPV behaviors by using 14 parallel items for fathers and mothers. For each item, participants report their answers on a 5-point Likert-type scale ranging from 0 (never) to 4 (very often = 6 times or more). Additionally, the measure includes eight parallel items exploring reactive and instrumental reasons that may motivate adolescents to commit CPV (Appendix B). 

#### 2.2.3. Social Information Processing in Conflicts with Parents Questionnaire (SIP)

This measure evaluates five components of social information processing: hostile attribution, anger, aggressive response access, anticipation of positive consequences of aggressive responses, and empathy. This scale was developed by Calvete [27] and consists of 21 items across three scenarios, with 7 items per scenario. Adolescents were instructed to imagine themselves in three distinct conflicts with their parents and to indicate how often they would respond in the ways described. Items were rated on a 5-point Likert scale ranging from 0 (never) to 4 (very often).

#### 2.2.4. Impulsive Subscale (IMP)

This subscale of the Cognitive and Social Strategies and Attitudes Questionnaire (AECS) [28] consists of seven items and assesses impulsivity using a 4-point Likert-type scale ranging from 1 (totally disagree) to 4 (totally agree).

### 2.3. Procedure

The research process began by contacting a team of four native specialists (psychology, family studies, psychometrics, and linguistics) to review the instruments and evaluate whether linguistic adaptation was necessary, according to the International Test Commission [29]. We analyzed the information gathered on construct content, linguistics, and grammar to inform adaptation decisions regarding questionnaire items.

For the CPV-Q, item 6 was adapted from “I have demanded my parents to buy me things even knowing they cannot afford it” to “I have demanded that my parents buy me things, even though they cannot pay” to improve clarity. For the reasons subscale, Item 6 was adapted from “Because of your own temper” to “Because of the way they are” for conciseness.

This study utilized probability-based stratified sampling with proportional allocation according to the population size across two provinces located in the south of Ecuador (Cañar and Cuenca). The sample size was calculated using the formula for finite populations at a 99% confidence level with a 3% margin of error. A probability-proportional stratified sampling design was used with fully random participant selection. The inclusion criteria were an age of 12–18 years, Ecuadorian citizenship/one-year residency minimum, and provision of informed assent and parental consent, which was explained upon data collection.

According to data from the Ministry of Education of Ecuador in 2023: 110,949 adolescents aged 12 and 18 had access to the National Education System: 85,178 in Azuay and 25,771 in Cañar. The following formula was used to calculate the sample size: $n=\frac{N*Z\frac{2}{\alpha }\;*\;p*q\;}{e^{2}*\left(N-1\right)+Z\frac{2}{\alpha }\;*p\;*\;q}$. The selection of schools and participants was random in both provinces; therefore, this study is representative for these populations.

Data were gathered in designated classrooms at educational institutions in the Azuay and Cañar provinces. This phase began when the decision of the Human Research Ethics Committee was favorable. Instruments were administered to the groups for 30–60 min. To guarantee the conditions for evaluation, the collaborators of this study were trained in the guidelines for administering the questionnaire. Standardized instructions were used to complement this process. All procedures performed in this study involving human subjects were in accordance with the ethical standards of the Ethics Committee for Human Studies of the University of Cuenca (2022-017E0-IE) and the 1964 Declaration of Helsinki and its later amendments or comparable ethical standards. This study was authorized and supervised by the Ministry of Education of Ecuador (Nro. MINEDUC-SFE-2022-00508-M)

### 2.4. Data Analysis

The analyses were performed with R software version 4.1 [30]. Before administering factorial treatment, we first conducted data screening to assess the distribution of the data and the assumptions required for factorial treatment. The multivariate normality of the data was analyzed using the Mardian test. To test these assumptions, we analyzed the residuals resulting from subjecting our data to linear regression with a set of random numbers. If the distribution of the residuals from a spurious regression showed anomalies, this could be because of our data. To analyze the structural validity of the scales, we conducted a confirmatory factor analysis (CFA) using the R package lavaan version 0.6-12 [31]. Owing to the absence of multivariate normality in our data, we used a diagonally weighted least-squares estimator. The reliability of the data was analyzed using Cronbach’s α and McDonald’s ω [32].

Validity tests were conducted by examining the correlations between the factor scores on the CPV-Q and the scale scores on the dimensions of the SIP and the impulsivity subscales. Previous studies have found evidence that CPV is related to the components of social information processing and impulsivity [2,20,24].

The SemTools package was used to evaluate convergent and discriminant validity [33]. The average variance extracted (AVE),used to evaluate convergent validity, had to have values equal to or greater than 0.37 [34]. Discriminant validity was assessed by calculating the heterotrait–monotrait (HTMT) ratio based on the mean of the correlations between items and factors, using a criterion of less than 0.85, to indicate discriminant validity [35].

Invariance analyses were performed using the lavaan R package [31]. Configural, metric, scalar, and strict invariances were tested. The criteria of Chen [26] were used to analyze invariance. This analysis consisted of observing whether the increase in the comparative fit index (CFI) was less than 0.010 and the increase in the root mean squared residuals of approximation (RMSEA) was less than 0.015.

Prevalence was calculated using IBM SPSS V29 software (New York, United States) (license: 425 cde720499d72b44ee). To analyze the prevalence of CPV, the percentages of behaviors reported by adolescents against their fathers and mothers were calculated. The association between the prevalence of CPV and the sex of the children and parents was analyzed using the *χ*^2^ and phi tests. The scores were recorded on the basis of two criteria. The first criterion was for those who had committed violent behavior on “at least 1 occasion”, and the second criterion of “reiterated violence” was for those who had committed violent behavior on two or more occasions. To analyze the reasons for the CPV, sex differences were calculated with a t-test for independent samples, calculating the effect size using Cohen’s d for fathers and mothers according to the sex of the children.

## 3. Results

### 3.1. Descriptive Statistics

The mean scores for the items analyzed in the scales were very low, with little variability in the scores (highest score, *M* = 0.694, *SD* = 0.961; see Table 1 and Table 2). These low scores have been common in other studies focusing on the general population [20,24]. The multivariate normality analysis showed that our data did not have a multivariate normal distribution. The correlation between item scores showed that our data met the assumptions of multicollinearity (*r* > 0.90), and singularity (*r* > 0.95). Analysis of the residuals resulting from running a regression with the data obtained through our scales and random numbers showed that our data met the assumptions of linearity, homogeneity, and homoscedasticity (most standardized regression residuals were between −2 and +2).

Table 1 and Table 2 show the standardized loadings shown by item, as well as the factors evaluated for both scales referring to fathers (CPV-FA and R-FA) and mothers (CPV-MO and R-MO, Table 2). As can be seen, the general behavior of the items of the scales was quite good, significantly explaining part of the variance of the latent variable to which they belonged. Nevertheless, some items showed standardized loadings below 0.50 (e.g., CPV7 and CPV5 for both fathers and mothers).

### 3.2. Convergent Validity

The average variance extracted (AVE) presented values above 0.37 in the psychological, physical, and control/domain CPV factors on the father and mother scales. However, the CPV’s financial factor had lower values than expected on both scales.

Statistically significant correlations in the expected directions were found (*p* < 0.01) between the CPV-Q factors (psychological, physical, financial, and control/domain violence) and the key dimensions of the social information processing (hostile attribution, aggressive response access, anger, and anticipation of positive consequences) and impulsivity subscales (Appendix A).

### 3.3. Confirmatory Factor Analyses

To analyze the psychometric properties of the scales used, we conducted CFA on each scale. The results showed an excellent fit for each of the scales: *χ*^2^ (71) = 102.269, *p* = 0.009, with CFI = 0.992, TLI = 0.990, SRMR = 0.064, RMSEA = 0.021 (RMSEA 90% CI [0.011, 0.030]) for CPV-F with reliability ratings of α = 0.78, G6 = 0.80, and ω = 0.82; *χ*^2^ (71) = 124.124, *p* = 0.000, with CFI = 0.988, TLI = 0.985, SRMR = 0.057, RMSEA = 0.024 (RMSEA 90% CI [0.017, 0.031]) for CPV-M with reliability ratings of α = 0.74, G6 = 0.75, and ω = 0.77; *χ*^2^ (19) = 50.408, *p* = 0.000, with CFI = 0.984, TLI = 0.977, SRMR = 0.057, RMSEA = 0.041 (RMSEA 90% CI [0.027, 0.055]) for reason-F with reliability ratings of α = 0.72, G6 = 0.70, and ω = 0.82; and, finally, *χ*^2^ (19) = 56.977, *p* = 0.000, with CFI = 0.987, TLI = 0.981, SRMR = 0.051, RMSEA = 0.039 (RMSEA 90% CI [0.028, 0.051]) for reason-M with reliability ratings of α = 0.74, G6 = 0.72, and ω = 0.83.

### 3.4. Discriminant Validity

The heterotrait–monotrait (HTMT) ratio values indicated adequate discriminant validity between the factors for both the father and mother scales (Table 3). For both scales, the values were lower than 0.85, and, in the subscale of reasons, the values were equally lower than 0.70 for both scales. The instrumental versus reactive reasons dimensions were compared.

### 3.5. Measurement Invariance by Sex

Table 4 presents the results of the CPV-Q invariance according to the sex of the child. The CPV-Q father scale revealed configural, metric, scalar, and strict invariance across sex in the Ecuadorian sample. However, the reasons subscale exhibited substantial differences by sex (CFI = 0.020; RMSEA = 0.016) at the scalar level. The CPV-Q mother scale revealed configural, metric, scalar, and strict invariance across sex in the Ecuadorian sample. Similarly, the reasons subscale demonstrated invariance.

### 3.6. Prevalence of Child-to-Parent Violence

Table 5 shows the prevalence of the types of CPV (psychological, physical, financial, and control/domain), according to the sex of the children and parents. Daughters had higher percentages of control/domain behaviors toward their father and mother, and we found a higher percentage of daughters than sons in psychological violence toward the mother. These differences were found both with the criterion of “at least 1 occasion” and with the criterion “reiterated violence”.

### 3.7. Mean CPV Ratio Scale Scores

The reasons used by the sons for engaging in CPV are listed in Table 6. On the instrumental reasons scale, sons scored higher than daughters on item 1 for both fathers and mothers. On the reactive reasons scale, daughters scored higher on item 6 for fathers and mothers and on item 8 for mothers.

## 4. Discussion

The first objective of this study was to adapt and analyze the psychometric validity of the Child-to-Parent Violence Questionnaire (CPV-Q), adolescent version, and analyze its reliability and validity [24]. The main contribution of this study is providing an instrument with evidence of validity and reliability adapted to the population of Ecuadorian adolescents. This will allow us to identify and evaluate this problem and to propose future studies in which risk and protective factors can be identified. This study is a first contribution for professionals working with families and is key because it proposes a perspective for the development of evidence-based prevention and intervention research. This study is significant because it unearthed epidemiological data that highlight an as-yet-undiscovered problem.

The structure of the four CPV factors represents the structure of the CPV-Q through the psychological, physical, economic, and control/domain aspects, together with a two-factor subscale comprising instrumental and reactive dimensions. The Ecuadorian sample demonstrated excellent goodness-of-fit, robust psychometric properties, and high internal consistency. These positive results mirror the findings of previous studies that examined CPV-Q among adolescent samples in Spain [24] and Chile [20].

Therefore, the first piece of evidence supports the first hypothesis. Confirmatory factor analysis indicated an excellent model fit and appropriate reliability thresholds. However, items 5 and 7 exhibited low factor loadings (<0.500). These items also showed minimal score variability and low values (maximum *M* = 0.694, *SD* = 0.961). These findings align with those of previous research on community-based populations, which have found comparably low variation and limited endorsement of these specific items [20,24].

Average variance extracted (AVE) showed acceptable values in the psychological, physical, and control/domain CPV factors on both the father and mother scales [34]. However, the CPV’s financial factor presented deficient values in this sample and should be reviewed in future research [20,24]. These findings imply that the financial and control/domain CPV factors should be interpreted with caution because they explained less than 50% of the variance, while the psychological and physical factors explained more than 50% of the variance.

The subscales of the reasons incorporated in the CPV-Q consist of two dimensions: instrumental and reactive. This subscale demonstrated reliability and psychometric validity in this Ecuadorian adolescent sample. These results are congruent with those of the CPV-Q validation in Spanish [24] and Chilean adolescents [20].

It also provides evidence of validity similar to those reported by Contreras [24] and Jímenez-García [20]. Statistically significant correlations were found between its factors, the key dimensions of the social information processing (SIP) scale, and the impulsivity subscale in most cases [20,24]. The heterotrait–monotrait (HTMT) ratio indicated adequate discriminant validity in all cases. Moreover, the assessment of discriminant validity through the heterotrait–monotrait (HTMT) ratio and convergent validity through the average variance extracted (AVE) represents a novel contribution that was lacking in previous validation studies of the CPV-Q.

The second objective was to demonstrate the invariance between the CPV-Q and the reason subscales. The CPV-Q parent scale permitted valid comparisons across adolescent sons and daughters, with equivalent items. However, the reasons subscale and measurement equivalence did not hold, precluding strict statistical comparisons by sex, while on the mother CPV-Q scale and the reasons subscale, all comparisons were valid due to compliance with all levels of invariance, thus demonstrating their equivalence. However, as this is the first contribution in this population, the data should be treated with caution until there is more evidence of noninvariance.

Thus, Hypothesis 2 is partially supported. Invariance testing revealed full metric, scalar, and strict invariance across sexes for both the CPV scale and the reasons subscale when applied to mothers. However, the father CPV scale demonstrated invariance, while the reasons subscale showed metric noninvariance, with CFI values above 0.010 and RMSEA values above 0.015 [26]. This suggests the need for additional invariance testing for the father reasons subscale, because the reasons for CPV perpetrated by sons and daughters may differ. Nonetheless, this noninvariance finding should be interpreted cautiously, pending further CPV-Q measurement equivalence studies across adolescent sex.

The third objective was to examine the frequency of different types of CPV, the reasons for violence, and associations with the sex of both parents and children. Psychological violence was the most prevalent form, with a high percentage of adolescents engaging in this behavior: 41.3–47.9% of the children had exercised this type of violence on at least one occasion, and 19.6–25.2% had reiterated this type of violence. These findings are similar to those of other studies conducted in Chile that placed this type of reiterated violence from 25.2–28.8% [20]; however, these figures are lower than those in European countries, where this type of violence can range from 28.8 to 91.5% [9,10].

Regarding physical violence, 5.1–6.8% of adolescents reported physically attacking their parents on at least one occasion, and 2–2.3% reported reiterated physical violence. Compared with a Chilean study that found rates from 2.5 to 5.4% for reiterated physical violence [20], the present results indicate a lower prevalence of reiterated physical violence against parents in this sample.

Financial violence was prevalent, from 37.9 to 42.8% on at least one occasion; 16.9–17.5% of adolescents reported reiterated financial violence. In comparison, Chilean research found markedly lower rates of 11.1–12.9% [20]. The control/domain was exercised by 53.0–62.8% on at least one occasion and by 30.5–35.5% for reiterated violence. Daughters demonstrated higher frequencies of control/domain behaviors directed toward both mothers and fathers than sons, aligning with the analogy previously noted in Chile [20].

Sons scored higher than daughters on item 1 of the instrumental reasons for fathers and mothers, and daughters scored higher than sons on items 6, 7, and 8 of the reactive reasons for mothers. These findings are partially similar to those of the study by Jiménez García [20]. These findings lay the groundwork for future research exploring the role of culture in the manifestations of CPV, taking the aspect raised in other contexts of interpersonal violence, such as violence in dating relationships, as a reference [36].

These findings provide evidence regarding the sex of the aggressor and the victim. In this particular study, there were differences in psychological violence toward the mother; daughters reported higher percentages than sons both when it occurred on at least one occasion and when it was reiterated. The control/domain behaviors were different: daughters presented higher percentages than sons toward the mother, both when it was at least on one occasion.

Consequently, Hypothesis 3 was partially supported, as differences were found. Psychological violence showed higher scores when daughters perpetrated this type of violence against their mothers, whether it was committed on at least one occasion or reiterated. Differences were also found in the control/domain toward the mother, with higher scores for daughters toward the mother when the behaviors were committed on at least one occasion. Daughters also had higher reactive ratio scores.

### Strengths and Weaknesses

The main strength of this research is the probabilistic design used to obtain the sample and the random selection of participants, which allowed us to include participants from different geographical areas with particularities in each place. One of the limitations of this study is its high experimental mortality rate. The analyses incorporated 62.33% (*n* = 1299) of the recruited sample in the analyses for the mother scale and 47.55% (*n* = 991) in the analyses for the father scale, as participants lacking contact with either parent in the past year, those with deceased parents, or those who had never met their father or mother were excluded from the analyses. Cases with high social desirability were also eliminated. Therefore, the findings may not be generalizable to the broader adolescent population.

While probability-based stratified sampling was used, the participants originated from only two southern Ecuadorian provinces (Cañar and Azuay). This limits the possibility of generalizing our results to the Ecuadorian adolescent population. Therefore, in future studies, we intend to incorporate samples from different provinces. Furthermore, this study is the first attempt to explore this problem in this population, so these results are based on adolescent self-reports; future studies should investigate the parents’ perspective.

Despite these limitations, this study contributes to the literature in several ways. First, it provides preliminary evidence supporting the validity and reliability of the CPV-Q in assessing CPV among Ecuadorian adolescents. In addition, high prevalence rates of CPV were found, which may indicate trajectories of escalation, underscoring the need for further research in this population.

In this study, statistically significant differences were found for psychological violence perpetrated against mothers, with daughters exhibiting a higher likelihood of this type of violent behavior more than twice. Similarly, regarding control/domain behaviors, daughters showed a higher probability of exerting control and domain actions on both fathers and mothers more than twice. In addition, daughters had a higher prevalence of reactive reasons.

Future CPV-Q refinement efforts should examine potential revisions to address the low factor loadings evidenced for items 5 “At home, we watch what I want on TV” and 7 “I have acquired debts that my parents have had to pay” to improve the discriminant validity of the financial violence and control/domain factors. Qualitative enquiry could elucidate whether item 5, assessing adolescents’ control/domain of television content choice, sufficiently reflects the current manifestations of parental control/domain, or whether alternate mechanisms of control and domain should be incorporated. The integration of qualitative methodologies may aid in measuring the relevance and specificity of the existing scale indicators.

This is the first step in the investigation of CPV in Latin America, considering that the evidence of the validity of the instruments for assessing CPV is indispensable and is required before a diagnosis of reality. However, future research is intended to cover other demands, such as the role of parental attributions, given that negative attributions are more frequent in parents whose children exhibit behavioral problems, which could be similar in parents suffering from CPV, and would be a clue for predicting violent behaviors toward parents.

Additional invariance testing research offers opportunities to evaluate the observed differences across adolescent sex in the reasons underlying CPV. Parent-perspective investigations would also significantly advance our understanding of this intricate issue’s developmental trajectories and the contributing contextual factors within families. Ultimately, multifaceted research programs are needed to elucidate the complex interplay between CPV among Ecuadorian adolescents and its psychosocial implications.

## Figures and Tables

**Table 1:** Factor loading for the father model of the Child-to-Parent Violence Questionnaire and reasons.

Factor	Item	*M*	*SD*	Skew	Kurt	Min	Max	Load	$\alpha_{O}$	ω	AVE
PSY	FA 1	0.206	0.512	3.194	13.497	0	4	0.669	0.825	0.712	0.558
	FA 2	0.316	0.610	2.245	5.967	0	4	0.748			
	FA 3	0.272	0.581	2.663	9.196	0	4	0.819			
	FA 4	0.111	0.440	5.090	30.765	0	4	0.744			
PHY											
	FA 8	0.054	0.279	6.676	59.289	0	4	0.840	0.916	0.712	0.802
	FA 10	0.017	0.151	9.855	106.389	0	2	0.925			
	FA 11	0.020	0.148	7.754	65.230	0	2	0.919			
FIN											
	FA 6	0.231	0.541	2.818	9.999	0	4	0.661	0.568	0.401	0.313
	FA 7	0.139	0.413	3.175	10.312	0	3	0.479			
	FA 12	0.263	0.554	2.474	7.651	0	4	0.522			
CD											
	FA 5	0.694	0.961	1.471	1.865	0	4	0.328	0.662	0.370	0.375
	FA 9	0.218	0.540	3.035	11.109	0	4	0.676			
	FA 13	0.047	0.248	6.538	54.042	0	3	0.807			
	FA 14	0.319	0.655	2.535	7.726	0	4	0.532			
IRs											
	R-FA 1	0.161	0.427	2.849	8.774	0	3	0.451	0.761	0.645	0.414
	R-FA 2	0.192	0.458	2.568	7.210	0	3	0.724			
	R-FA 3	0.308	0.519	1.556	2.281	0	3	0.742			
	R-FA 4	0.294	0.568	2.133	5.013	0	3	0.671			
	R-FA 5	0.123	0.401	3.986	19.158	0	3	0.585			
RRs											
	R-FA 6	0.379	0.683	2.026	4.116	0	3	0.665	0.793	0.661	0.604
	R-FA 7	0.091	0.339	4.316	21.584	0	3	0.803			
	R-FA 8	0.205	0.502	2.779	8.550	0	3	0.852			

Note: Father sample, 991; PSY, psychological; PHY, physical; FIN, financial; CD, control/domain; IRs, instrumental reasons; RRs, reactive reasons; *M*, median; *SD*, standard deviation; Skew, skewness; Min, minimum; Max, maxim; Load, factor loadings; $\alpha_{O}$, alpha ordinal; ω, McDonald’s; AVE, average variance extracted.

**Table 2:** Factor loading for the mother model of the Child-to-Parent Violence Questionnaire and reasons.

Factor	Item	*M*	*SD*	Skew	Kurt	Min	Max	Load	$\alpha_{O}$	ω	AVE
PSY	MO 1	0.223	0.542	3.122	12.652	0	4	0.696	0.809	0.704	0.535
	MO 2	0.323	0.614	2.228	6.088	0	4	0.711			
	MO 3	0.285	0.565	2.247	6.086	0	4	0.852			
	MO 4	0.140	0.453	4.233	22.515	0	4	0.652			
PHY											
	MO 8	0.059	0.260	5.038	30.236	0	3	0.769	0.879	0.615	0.722
	MO 10	0.016	0.149	11.66	171.217	0	3	0.882			
	MO 11	0.024	0.162	7.293	58.112	0	2	0.893			
FIN											
	MO 6	0.227	0.500	2.296	5.318	0	3	0.589	0.585	0.409	0.317
	MO 7	0.149	0.447	3.597	15.970	0	4	0.525			
	MO 12	0.334	0.634	2.462	8.103	0	4	0.573			
CD											
	MO 5	0.664	0.957	1.459	1.654	0	4	0.404	0.688	0.428	0.375
	MO 9	0.201	0.531	3.391	14.715	0	4	0.671			
	MO 13	0.054	0.255	5.703	41.142	0	3	0.723			
	MO 14	0.341	0.679	2.581	8.299	0	4	0.603			
IRs											
	R-MO 1	0.177	0.449	2.820	9.025	0	3	0.575	0.783	0.656	0.429
	R-MO 2	0.193	0.458	2.596	7.670	0	3	0.693			
	R-MO 3	0.295	0.522	1.725	3.035	0	3	0.751			
	R-MO 4	0.355	0.619	1.891	3.778	0	3	0.634			
	R-MO 5	0.163	0.457	3.256	12.047	0	3	0.605			
RRs											
	R-MO 6	0.413	0.699	1.880	3.478	0	3	0.700	0.813	0.687	0.618
	R-MO 7	0.103	0.375	4.200	19.866	0	3	0.803			
	R-MO 8	0.236	0.543	2.580	7.133	0	3	0.848			

Note: Mother sample, 1299; PSY, psychological; PHY, physical; FIN, financial; CD, control/domain; IRs, instrumental reasons; RRs, reactive reasons; *M*, median; *SD*, standard deviation; Skew, skewness; Min, minimum; Max, maxim; Load, factor loadings; $\alpha_{O}$, alpha ordinal; ω, McDonald’s; AVE, average variance extracted.

**Table 3:** Discriminant validity of the CPV-Q and the reasons for the father and mother scale.

	Father				Mother			
	PSY	PHY	FIN	CD	PSY	PHY	FIN	CD
PSY								
PHY	0.519				0.434			
FIN	0.471	0.542			0.518	0.429		
CD	0.609	0.406	0.849		0.612	0.382	0.761	
	RI	RR			RI	RR		
IRs								
RRs	0.585				0.537			

Note: Sample fathers, 991; sample mothers, 1299; PSY, psychological; PHY, physical; FIN, financial; CD, control/domain; IRs, instrumental reason; RRs, reactive reason.

**Table 4:** Fit indices for measurement invariance by child’s sex for father ratings.

	Father										
Model	$x^{2}$		$x^{2}/df$	CFI	ΔCFI	RMSEA	LOW	UPP	ΔRMSEA	SRMR	ΔSRMR
CPV-F											
Configural	89.232	(142)	0.628	0.999	0.000	0.000	0.000	0.000		0.063	
Metric	138.141	(152)	0.909	0.999	0.000	0.000	0.000	0.015	0.000	0.079	0.016
Scalar	150.011	(162)	0.926	0.999	0.000	0.000	0.000	0.016	0.000	0.080	0.001
Strict	159.299	(176)	0.905	0.999	0.000	0.000	0.000	0.013	0.000	0.089	0.009
REA-F											
Configural	38.122	(38)	1.000	0.999		0.003	0.000	0.032		0.058	
Metric	44.309	(44)	1.000	0.999	0.000	0.004	0.000	0.030	0.001	0.060	0.002
Scalar	59.898	(50)	1.197	0.979	0.020 *	0.020	0.000	0.037	0.016 *	0.063	0.003
Strict	72.128	(58)	1.244	0.970	0.009	0.022	0.000	0.037	0.002	0.083	0.020
	**Mother**										
**Model**	$x^{2}$ **(*df*)**		$x^{2}/df$	CFI	ΔCFI	RMSEA	LOWE	UPPER	ΔRMSEA	SRMR	ΔSRMR
CPV-M											
Configural	115.722	(182)	0.636	0.999	0.000	0.000	0.000	0.002	0.000	0.052	
Metric	143.321	(152)	0.942	0.999	0.000	0.000	0.000	0.015	0.000	0.061	0.009
Scalar	155.532	(162)	0.960	0.999	0.000	0.000	0.000	0.016	0.000	0.061	0.000
Strict	167.950	(176)	0.954	0.999	0.000	0.000	0.000	0.015	0.000	0.078	0.017
REA-M											
Configural	36.334	(38)	0.956	0.999	0.000	0.000	0.000	0.026	0.000	0.046	
Metric	40.969	(44)	0.931	0.999	0.000	0.000	0.000	0.023	0.000	0.049	0.003
Scalar	44.653	(50)	0.893	0.999	0.000	0.000	0.000	0.021	0.000	0.050	0.001
Strict	52.259	(58)	0.901	0.999	0.000	0.000	0.000	0.020	0.000	0.060	0.010

Note: Sample fathers, 991; sample mothers, 1299; CPV-F = Child-to-Parent Violence Questionnaire, father; CPV-M = Child-to-Parent Violence Questionnaire, mother; REA-F = reasons, father; REA-M = reasons, mother; *x*^2^ = normed chi-square; *df*, degrees of freedom; *CFI* = comparative fit index; *RMSEA* = root mean-square error of approximation; *SRMR* = standardized root mean square residual; * violation of measurement invariance.

**Table 5:** Prevalence of child-to-parent violence by sex of parents and children.

	Father				Mother			
	Sons (%)	Daughters (%)	$x^{2}$	φ	Sons (%)	Daughters (%)	$x^{2}$	φ
At least 1 occasion								
PSY	42.7	41.9	0.036	0.008	41.3	47.5	4.725 *	0.062
PHY	6.1	5.1	0.337	0.023	6.8	6.7	0.000	0.002
FIN	38.9	37.9	0.055	0.010	41.6	42.8	0.135	0.012
CD	54.3	62.8	6.922 **	0.086	53.0	59.3	4.937 *	0.063
Reiterated violence								
PSY	20.0	20.0	0.000	0.000	19.6	25.2	5.388 *	0.066
PHY	2.0	2.0	0.000	0.002	2.3	1.8	0.176	0.017
FIN	17.0	15.1	0.577	0.027	17.5	16.9	0.037	0.007
CD	30.5	35.5	2.111	0.048	31.8	34.0	0.579	0.023

Note: Sample fathers, 991; sample mothers, 1299; PSY, psychological; PHY, physical; FIN, financial; CD, control/domain. The correlation coefficient was * *p* < 0.05, ** *p* < 0.01.

**Table 6:** Reasons for child-to-parent violence by sex of parents and children.

	Father				Mother			
	Sons *n* = 440 *M* (*SD*)	Daughters *n* = 551 *M* (*SD*)	t	d	Sons *n* = 572 *M* (*SD*)	Daughters *n* = 727 *M* (*SD*)	t	d
Instrumental								
1	0.22 (0.489)	0.12 (0.363)	3.645 **	0.424	0.20 (0.487)	0.16 (0.416)	1.556	0.448
2	0.18 (0.434)	0.20 (0.476)	−0.756	0.458	0.19 (0.454)	0.19 (0.462)	0.058	0.458
3	0.31 (0.522)	0.31 (0.518)	−0.051	0.520	0.30 (0.508)	0.29 (0.534)	0.038	0.522
4	0.33 (0.586)	0.27 (0.553)	1.544	0.568	0.35 (0.594)	0.36 (0.638)	−0.364	0.619
5	0.14 (0.425)	0.11 (0.380)	1.235	0.401	0.18 (0.457)	0.15 (0.458)	0.935	0.457
Reactive								
6	0.35 (0.679)	0.40 (0.685)	−1.120	0.683	0.33 (0.638)	0.48 (0.738)	−3.879 **	0.696
7	0.09 (0.352)	0.09 (0.329)	0.008	0.339	0.08 (0.345)	0.12 (0.396)	−1.670 *	0.374
8	0.18 (0.455)	0.23 (0.536)	−1.445	0.502	0.18 (0.442)	0.28 (0.607)	−3.235 **	0.541

Note. Sample fathers, 991; sample mothers, 1299. The correlation coefficient was * *p* < 0.05, ** *p* < 0.001.

**Figure A1:**
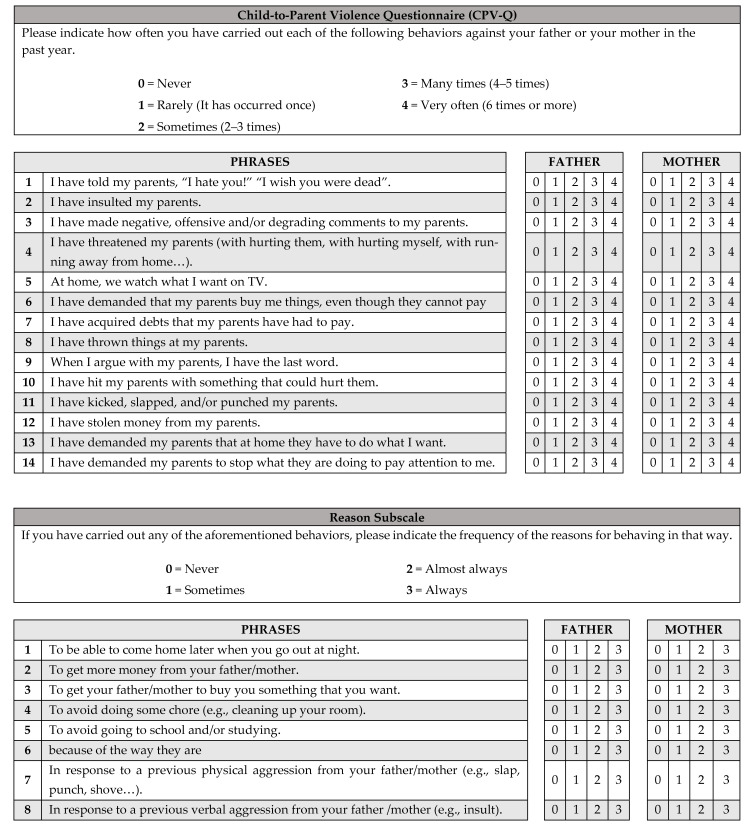
Child-to-Parent Violence Questionnaire (CPV-Q).

## Data Availability

The data supporting this research are open access. They can be accessed by sending an email to the first author of this article.

## Supplementary Materials

The following supporting information can be downloaded at: https://www.mdpi.com/article/10.3390/children11070845/s1, File S1: Child-to-Parent Violence Questionnaire (CPV-Q), Spanish Version.

## Appendix A

Table A1 presents the external validity of the CPV-Q with the SIP scale and the impulsivity subscale. These results were similar in other studies [20,24].

**Table A1:** Bivariate correlations of the Child-to-Parent Violence Questionnaire (CPV-Q) and the dimensions of social information processing and the impulsiveness scale (SIP).

	Father/Mother										
	1	2	3	4	5	6	7	8	9	10	11
PSY	--	0.316 **	0.328 **	0.313 **	0.286 **	0.479 **	0.186 **	0.261 **	0.186 **	0.049	0.263 **
PHY	0.342 **	--	0.313 **	0.189 **	0.210 **	0.251 **	0.056 *	0.080 **	0.207 **	0.147 **	0.089 **
FIN	0.310 **	0.304 **	--	0.345 **	0.417 **	0.287 **	0.175 **	0.246 **	0.156 **	0.131 **	0.230 **
CD	0.298 **	0.206 **	0.338 **	--	0.335 **	0.299 **	0.158 **	0.281 **	0.161 **	0.093 **	0.203 **
IR	0.297 **	0.241 **	0.403 **	0.362 **	--	0.351 **	0.224 **	0.258 **	0.192 **	0.138 **	0.196 **
RR	0.446 **	0.286 **	0.279 **	0.291 **	0.357 **	--	0.235 **	0.319 **	0.185 **	0.037	0.296 **
HA	0.170 **	0.035	0.146 **	0.150 **	0.199 **	0.211 **	--	0.327 **	0.242 **	0.229 **	0.251 **
AN	0.236 **	0.079 **	0.253 **	0.245 **	0.234 **	0.291 **	0.327 **	--	0.328 **	0.056 *	0.327 **
ARA	0.182 **	0.225 **	0.160 **	0.164 **	0.213 **	0.198 **	0.242 **	0.328 **	--	0.417 **	0.118 **
APC	0.077 **	0.085 **	0.105 **	0.081 **	0.131 **	0.049	0.229 **	0.056 *	0.417 **	--	0.056 *
IMP	0.246 **	0.111 **	0.213 **	0.179 **	0.166 **	0.288 **	0.251 **	0.327 **	0.118 **	0.056 *	--

Note. PSY: psychological; PHY: physical; FIN: financial; CD: control/domain; HA: hostile attribution; AN: anger; ARA: aggressive response access; APC: anticipation of positive consequences; IMP: impulsivity. Values in square brackets indicate the 95% confidence intervals for each correlation. The confidence interval is a plausible range of population correlations that could have caused sample correlation (Cumming, 2014). **, The correlation is significant at the 0.01 level (bilateral); *, The correlation is significant at the 0.05 level (bilateral).

## Appendix B

Figure A1 shows the CPV-Q and the reasons subscale used in this study.
