# Supplementary material for: Assessment of Adolescents in Child-to-Parent Violence: Invariance, Prevalence, and Reasons
Source: Children (Basel). 2024 Jul 12;11(7):845. doi: 10.3390/children11070845 (PMC11275027; doi:10.3390/children11070845)
Supplement: Supplementary file 1 [file children-11-00845-s001.zip › children-3076047-supplementary.pdf]

Cuestionario de Violencia Filio-Parental (C-VIFIP)

Instrucciones: A continuación, encontrarás una serie de frases que describen conductas que los hijos/as pueden mostrar hacia los padres y madres en determinados momentos, sobre todo en la adolescencia, etapa en la que esas relaciones pueden ser conflictivas y desembocar en discusiones y disputas. Por favor, lee detenidamente cada frase e indica con qué frecuencia has llevado a cabo cada una de las siguientes conductas hacia tu padre y/o tu madre **DURANTE EL ÚLTIMO AÑO**. Marque con una X.

0 = Nunca

1 = Raramente (Ha ocurrido alguna vez)

2 = Algunas veces (2-3 veces)

3 = Bastantes veces (4-5 veces)

4 = Muy a menudo (6 veces o más)

	FRASES	PADRE					MADRE				
1	He llegado a decir cosas a mis padres como "¡te odio!", "¡ojalá te mueras!".	0	1	2	3	4	0	1	2	3	4
2	He insultado a mis padres.	0	1	2	3	4	0	1	2	3	4
3	He hecho comentarios negativos, ofensivos y/o degradantes a mis padres.	0	1	2	3	4	0	1	2	3	4
4	He amenazado a mis padres (con hacerles daño, con hacerme daño a mí mismo/a, con irme de casa...).	0	1	2	3	4	0	1	2	3	4
5	En casa, vemos lo que yo quiero en la televisión.	0	1	2	3	4	0	1	2	3	4
6	He exigido a mis padres que me compren cosas, incluso sabiendo que no lo podrían pagar.	0	1	2	3	4	0	1	2	3	4
7	He adquirido deudas que mis padres han tenido que pagar.	0	1	2	3	4	0	1	2	3	4
8	He lanzado cosas a mis padres.	0	1	2	3	4	0	1	2	3	4
9	Cuando discuto con mis padres, yo tengo la última palabra.	0	1	2	3	4	0	1	2	3	4
10	He dicho cosas negativas y ofensivas a mis padres.	0	1	2	3	4	0	1	2	3	4
11	He golpeado a mis padres con algo que pudiera hacerles daño.	0	1	2	3	4	0	1	2	3	4
12	He dado una patada, bofetada o puñetazo a mis padres.	0	1	2	3	4	0	1	2	3	4
13	He robado dinero a mis padres.	0	1	2	3	4	0	1	2	3	4
14	He exigido a mis padres que en casa se haga lo que yo quiera.	0	1	2	3	4	0	1	2	3	4
15	He exigido a mis padres que dejen lo que estaban haciendo para que me atiendan.	0	1	2	3	4	0	1	2	3	4

Razones

Si has llevado a cabo algunas de las conductas anteriores, por favor, indica la frecuencia de las **RAZONES** por las que crees que has actuado de ese modo. Si no has llevado a cabo ninguna de las conductas anteriores, **SELECCIONA LA OPCIÓN NUNCA (0).**

0 = Nunca

1 = Algunas veces

2 = Casi siempre

3 = Siempre

	**FRASES**	**PADRE**				**MADRE**			
**1**	Por querer llegar más tarde a casa cuando sales por la noche.	**0**	**1**	**2**	**3**	**0**	**1**	**2**	**3**
**2**	Para que tu padre/madre te dé más dinero.	**0**	**1**	**2**	**3**	**0**	**1**	**2**	**3**
**3**	Para que tu padre/madre te compre algo que quieres.	**0**	**1**	**2**	**3**	**0**	**1**	**2**	**3**
**4**	Para evitar hacer alguna tarea (p.ej., limpiar tu habitación).	**0**	**1**	**2**	**3**	**0**	**1**	**2**	**3**
**5**	Para evitar ir a clase y/o estudiar.	**0**	**1**	**2**	**3**	**0**	**1**	**2**	**3**
**6**	Por tu forma de ser.	**0**	**1**	**2**	**3**	**0**	**1**	**2**	**3**
**7**	En respuesta a una previa agresión física de tu padre/madre (p.ej., bofetada, puñetazo, empujón…).	**0**	**1**	**2**	**3**	**0**	**1**	**2**	**3**
**8**	En respuesta a una previa agresión verbal de tu padre/madre (p.ej., insulto).	**0**	**1**	**2**	**3**	**0**	**1**	**2**	**3**
